# Zinc, manganese and copper amino acid complexed in laying hens’ diets affect performance, blood parameters and reproductive organs development

**DOI:** 10.1371/journal.pone.0239229

**Published:** 2020-11-04

**Authors:** Camilla Gomes Pereira, Carlos Boa-Viagem Rabello, Mércia Rodrigues Barros, Helena Emilia C. C. C. Manso, Marcos Jose Batista dos Santos, Andresa G. Faria, Heraldo Bezerra de Oliveira, Waleska Rocha Leite Medeiros-Ventura, Rogerio Ventura Silva Júnior, Cleyton C. D. Carvalho, Alba K. Fireman

**Affiliations:** 1 Universidade Federal Rural de Pernambuco, Recife, Pernambuco, Brazil; 2 Zinpro Corporation, Eden Prairie, Minnesota, United States of America; Tokat Gaziosmanpasa University, TURKEY

## Abstract

In the intestinal lumen, excess of oxides and sulfates interfere with the absorption of minerals due to competition from the same absorption site. Amino acids-mineral complexed (AACM) is intended to minimize these problems, which might be absorbed by different absorption sites. Then, a study including Zinc (Zn), Manganese (Mn) and Copper (Cu) from different sources was carried out to evaluate the performance, blood parameters and reproductive organs development of Brown Laying Hens. A total of 800 Lohmann Brown Lite were fed, from one-day-old to 182-days-old, Zn, Mn and Cu from different sources. Measurements were made from 105 to 182-days-old. The laying hens were distributed according to a completely randomized design with 20 replicates and 20 birds per experimental unit. The treatments consisted of a diet supplemented with 70, 70 and 8 mg/kg of Zn, Mn and Cu; respectively, from inorganic sources (IM). The second treatment contained 40, 40 and 2.75 mg/kg of Zn, Mn and Cu, respectively from IM plus 30, 30 and 5.25 mg/kg of Zn, Mn and Cu; respectively, from AACM sources. Performance and reproductive organs development (oviduct and ovary weight), tibia weight, liver weight, egg output and body weight, and blood variables were evaluated. Data were compared by Student’s t-test (*P < 0*.*05*). Laying hens fed AACM reached 35% of egg output two days earlier and presented heavier tibia bone than the IM group. Those hens also presented greater oviduct weight, greater hematocrit and greater serum concentration of total leukocytes, erythrocytes, eosinophils, monocytes and the hormones T4 and FSH, than the hens fed IM. The supplementation of AACM in laying hens’ diets since one-day-old improves the productive performance from the beginning of egg output to peak production, which is justified by better development of bones and oviduct, hormone production and immune system support.

## Introduction

The improvement in egg production per hen housed is the most important selection criterion in layer breeding [1]. In addition to genetics, housing system and animal nutrition affect bone strength and eggshell strength [2, 3]. With an increased production cycle length, skeletal integrity and bone fracture in layers are gaining more importance. It is possible that the earlier preparation of medullary bones development in laying hens promote a longer productive life without consequences such as osteoporosis, bone fractures or cage fatigue. Most of the studies on nutritional effects on bone quality and, consequently, eggshell quality in laying hens have been focused on macro-minerals calcium (Ca), phosphorus (P) and vitamin D3. Although it is known, that trace-elements, such as zinc (Zn), manganese (Mn) and copper (Cu) play important roles as enzymatic systems cofactors related to the mineralization process, there is still a limited number of studies about the relationship between trace elements, bone quality and eggshell quality for laying hens.

The medullary bone acts as a labile reserve of Ca quickly mobilized when the eggshell is being calcified, and this mobilization is performed by cellular activity in the bone. Calcification of each shell is accompanied by bone resorption associated with intense osteoclastic activity, and soon after the first egg is laid the bone-resorbing phase gives way to one dominated by bone—synthesis [4].

The importance of bone mineralization processes in laying hens is aligned with the eggshell quality, which in turn, affects the egg industry. Cracked or broken eggs account for 80 to 90% of the eggs that are routinely downgraded. Eggshell is the package for the egg contents and is also the first barrier against bacterial penetration [5]. So, it must be free of fails in order to offer the best quality package for best quality internal contents. Mabe et al. [5] suggested that trace elements as Zn, Mn and Cu could affect mechanical properties of the eggshell by their effect on calcite crystal formation and modifying crystallographic structure of the eggshell.

The elements Zn, Mn and Cu are constituents of several proteins involved in intermediary metabolism, hormone secretion, and the immune system [6]. Currently, the industry includes inorganic sources (IM) of trace minerals in laying hens’ diets with levels mainly derived primarily from manuals. The recommendations do not consider the bioavailability of those trace minerals, which is known not to be similar.

In the intestinal lumen, excess of oxides and sulfates interfere with the absorption of minerals due to competition from the same absorption site [7], such as iron (Fe) and Mn, Ca and Zn or Zn and Cu. However, the synergism between some minerals also occurs when the supply is balanced. Even in synergism, these sources may become unavailable [7], as they may complex with products of the Maillard reaction, mainly Zn, Cu, Mg and Ca [8]. Also, phytates, inositol, hexa-phosphates and penta-phosphates bind to minerals reducing their availability [9, 10].

Thus, the concept of mineral bound to an organic molecule (OMM) is intended to minimize these problems, which might be absorbed by different absorption sites [11] which would avoid the ionic competition between ionized elements as with the oxides and sulfates salts [12]. However, there are different OMMs such as proteinates, glycinates, amino acids-mineral complexed (AACM), metal-MHA chelates, metal-polysaccharide complexes and metal-propionates [12], each one with different absorption capacity [13]. The use of OMMs in poultry feed can improve the immune response, reproduction and animal growth [14, 15], improving zootechnical indexes. It was observed a high percentage of egg output (EO) for the birds that fed diets with complexed minerals, compared with birds that received a diet with conventional sources of minerals during the second production cycle [16]. The diet with Zn, Mn and Cu as AACM improved the eggshell weight and thickness of broiler breeders’ eggs, without affecting egg weight (EW) [17]. Also, it was observed an increased eggshell breaking strength when the diets of laying hens were supplemented with Zn and Mn as AACM compared with diets containing Zn and Mn as oxides [18].

Using Caco-2 cells methodology, Gao et al. [19] showed that all the AACM forms facilitated Cu absorption. The apparent permeability of Cu ions in these complex forms, were at least 7.6-fold higher than those in the CuSO4 form. Using the same model [20], concluded that Zn AACM may possess an advantage over classical Zn supplements such as Zn salts, as it is able to increase Zn bioavailability. Due to its chemical form, AACM remains intact and does not suffer dissociation with low pH or enzymatic digestion in the gastrointestinal tract [21].

In the growing period, laying hen type birds undergo significant physiological changes to initiate the reproductive phase, demanding a high amount of nutrients for its physiological processes, bone and splenic tissue growth [22]. In several of these processes, trace minerals are needed. It is known that malnutrition at this stage causes irreversible damage in the productive phase, although the effect of AACM inclusion starting from the initial diets and the subsequent effect from the beginning of EO phase till the peak of production is unknown.

Thus, this study was carried out to evaluate the AACM (Zn, Mn, and Cu) on the performance and reproduction of semi-heavy laying hens until the peak phase. On the hypothesis that the use of AACM may influence the performance of semi-heavy laying hens.

## Materials and methods

The protocol for the accomplishment of this study was authorized by the Commission of Ethics in the use of animals—CEUA of the Federal Rural University of Pernambuco under the number of the license 064/2016.

### Bird husbandry

Three thousand one-day-old Lohmann Brown Lite laying hens were used and supplied from the first day of life with one of two experimental diets, with 1500 birds for each treatment. At 14 wk of age, the hens were individually weighed, and 800 hens were selected and placed in the cages, considering average weight and uniformity. Performance data was done from wk 15 to wk 26 of age. The birds were vaccinated against colibacillosis, Newcastle, Gumboro, Marek, infectious bronchitis, pneumovirus, infectious coryza, salmonella, Avipoxvirus, encephalomyelitis and Egg Drop Syndrome.

The hens were housed in a caged layer house with five hens per cage (50 cm x 40 cm x 45 cm), each cage was equipped with two nipple drinkers and one feeder, water and diets were ad libitum. The light program adopted was 12 hours of natural light until 18^th^ week and after 19^th^ week there was an increase of 30 min of artificial light per week until reaching 16 hours of total light.

**Fig 1:**
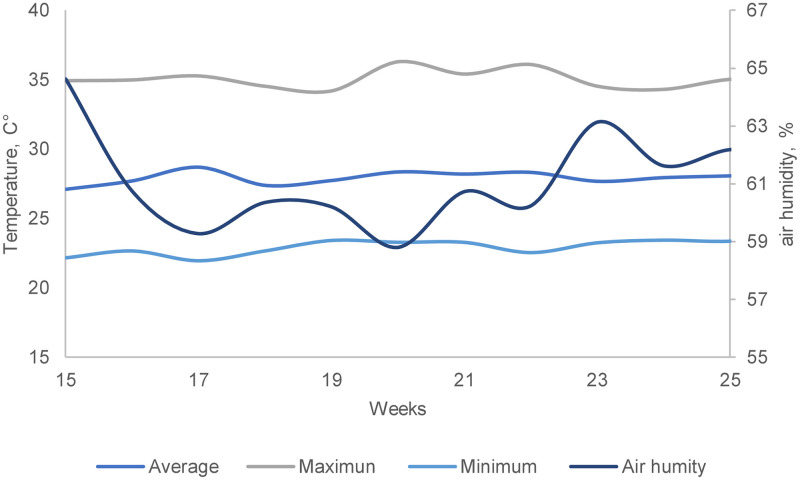
Mean variation of temperature (T, °C) and relative air humidity (%) during the ten weeks of the experimental period.

The temperatures and relative air humidity were recorded using digital thermo-hygrometers (Incoterm 7666.02.0.00), always at 10 a.m. Data Loggers (HOBO ware, Onset Company) were also installed for light intensity, temperature and relative humidity registration. Temperature and relative humidity data are shown in Fig 1.

### Design and experimental diets

The birds were distributed in a completely randomized design, with two treatments, each consisting of 20 replicates and 20 birds per replicates. Corn-soybean meal diets were formulated to contain adequate levels of all nutrients as recommended by Rostagno et al. [23] and Lohmann Brown Lite manual, and the treatments consisted of supplementation of these diets with 70, 70 and 8 mg/kg of inorganic Zn, Mn and Cu (IM) or 40, 40 and 2.75 mg/kg inorganic minerals associated with 30, 30 and 5.25 mg/kg of Zn, Mn and Cu as AACM; respectively. Vitamin and mineral premixes were formulated to meet the birds’ nutritional requirements, according to the levels commonly used in the industry of brown eggshell hens (Table 1).

**Table 1:** Trace mineral composition of treatment diets.

Trace Mineral*	IM	AACM	
	mg/kg	mg/kg	
	Inorganic^1^	Inorganic^1^	Availa ZMC^2^
Zinc	70	40	30
Manganese	70	40	30
Copper	8	2.75	5.75

^1^ Inorganic sources of zinc, manganese and copper were, respectively, ZnO, MnO and CuSO_4_.

^2^ The amino acid complex sources of zinc, manganese and copper were Availa^®^Zn, Availa^®^Mn and Availa^®^Cu (Zinpro Corp., Eden Prairie, MN, United States).

*The levels of Iron (FeSO_4_), Iodine (Ca(IO_3_)_2_) and Selenium (Na_2_SeO_3_) for both experimental diets were 50, 1 and 0.25 mg/kg; respectively. AACM—Amino acids-mineral complexed.

The IM were Zn oxide (73%), Mn oxide (57%), Cu sulfate (34.5%), ferrous sulfate (30% Fe), Ca iodate (62% I) and sodium selenite (45% Se). The Zn, Mn and Cu AACM source was Availa ZMC (Zinpro Corporation—Minnesota, USA). Three diets were provided, according to the physiological stage of hens (Table 2).

**Table 2:** Composition of the basal diet (%).

Ingredients	Phases		
	Growth (13-16wks)	Pre-Laying (17-20wks)	Peak of Production (21-26wks)
Corn	73.83	64.14	55.31
Soybean Meal	21.90	26.10	30.50
Soy oil	0.53	0.36	3.13
Salt	0.29	0.29	0.26
Calcium carbonate	1.18	3.58	8.80
Dicalcium phosphate 18.5%	1.45	0.89	0.92
Vitamin Premix^1^	0.10	0.10	0.10
Mineral Premix—Inorganic	0.10	0.10	0.10
Probiotic^2^	0.04	0.04	0.10
Mycotoxin binder^3^	0.20	0.20	0.20
Sodium bicarbonate	0.15	0.15	0.15
DL-Methionine 99	0.13	0.26	0.32
Mineral Premix—AACM	0.10	0.10	0.10
Phytase AB Vista4	0.01	0.01	0.01
Threonine 98,5	-	0.04	0.06
Kaolin	-	3.64	-
**Composition calculated**			
Metabolizable Energy (kcal/kg)	3038	2800	2850
Crude Protein (%)	15.69	17.00	18.40
Dry Matter (%)	88.74	87.32	89.02
Calcium (%)	0.97	2.00	3.80
Available Phosphorus (%)	0.45	0.45	0.47
Digestible Lysine (%)	0.71	0.80	0.90
Digestible Methionine (%)	0.36	0.50	0.58
Digestible Met. + Cist. (%)	0.58	0.73	0.82
Digestible Threonine (%)	0.53	0.61	0.68
Digestible Tryptophan (%)	0.16	0.18	0.20
Digestible Arginine (%)	0.94	1.04	1.16
Digestible Isoleucine (%)	0.66	0.71	0.77
Digestible Valine (%)	0.66	0.72	0.78
Chlorine (%)	1.75	1.50	2.71
Sodium (%)	0.18	0.18	0.17
Choline (mg/kg)	1069.81	1127.28	1194.54

^1^ Premix vitaminic provides: Vitamin A (min): 8,000,000 IU / kg, Vitamin D3 (min): 2,500,000 IU / kg, Vitamin E (min): 6,000 IU/kg, Vitamin K3 (min): 1,000 mg/kg, Vitamin B1 (min): 1000 mg/kg, Vitamin B2 (min): 4,500 mg/kg, Vitamin B6 (min): 2,000 mg/kg, Vitamin B12 (min) 12,000 mcg/kg, Niacin (min): 15 g/kg, Calcium Pantothenate (min): 6.000 mg/kg, Folic Acid (min): 400 mg/kg, Biotin (min): 25 mg/kg.

^2^ Calsporin^®^ BSG provides per kg of diet: *Bacillus subtillis*: 1 x 10E10 cfu/g.

^3^ Biobond provides per kg of diet: Hydrated sodium and calcium aluminosilicates: 1000 g/kg;

^4^ Phytase provides per kg of diet (min): 10,000 FTU/g. AACM—Amino acids-mineral complexed.

The mineral composition of water and diets fed during the experimental period was analyzed and the results are shown in Table 3.

**Table 3:** Minerals chemical analysis of water and diets.

Trace Mineral	Drinking water (mg/L)	Diets (mg/kg)					
		Growth		Pre-Laying		Peak of production	
		IM	AACM	IM	AACM	IM	AACM
Zn	<0.01	104.7	106.1	101	116	99	105.3
Mn	0.018	86	96.2	91	92.3	93	86
Cu	<0.01	16	18	13	17	12.2	15.1

The analysis was performed at the Soil Environmental Chemistry Laboratory of Universidade Federal Rural de Pernambuco.

IM—Inorganic Mineral, AACM—Amino acids-mineral complexed; P = Probability, SEM = Mean Standard Error.

### Data collection

The birds were fed the experimental diets since one-day-old and the whole period of data collection, which lasted 76 days, from week 15th to the 26th. In the period of 13 to 16 weeks, the bids fed growth diets, 17 to 20 weeks pre-laying diets and 21 to 26 weeks the laying hens fed production diets. Eggs were collected three times a day, at 9:00 am, 1:00 pm and 4:00 pm, and at the same time, they were counted and weighed. At the end of the experimental period, the birds were euthanatized through cervical dislocation in accordance with animal welfare standards. Afterward, an incision was proceeded in the abdominal cavity to collect the organs. Isthmus, magnus, uterus and ovary, beyond the liver and tibia (right and left) were collected and individually weighed, using a digital analytical balance with accuracy of 0.01 g (Bel, model L 3102iH). Tibias, after removal, were stored in plastic bags and frozen at -20°C for later analysis of bone composition and breaking strength.

### Performance data

The performance parameters evaluated were body weight (BW, kg), body weight gain (BWG, g), average daily feed intake (ADFI, kg), EO (%), egg mass (EM, g) and EW (g). The BWG was determined by the difference between the initial and final weights of each period; the ADFI by the difference between the ration supplied and the leftovers of the buckets and the feeders.

### Blood parameters

Blood collection was done through the jugular puncture, where approximately 4 ml of blood was collected in tube containing EDTA, turning twice slowly to homogenize the liquids. After centrifugation at 3800–4000 rpm for five minutes, the serum was obtained and, approximately, 1 ml of this serum was placed in the Eppendorf and frozen, which was later unfrozen and analyzed.

From the blood samples analyses of progesterone, follicle-stimulating hormone (FSH), luteinizing hormone (LH), thyroxine (T4), triiodothyronine (T3) and estradiol were performed. Blood serum samples were maintained in 1.5 ml microtubes each and stored at -80°C. For analysis, the samples were unfrozen at room temperature, homogenized in a vortex, centrifuged at a spin speed of 3000 G for 10 minutes. Subsequently, each sample was introduced for analysis in individual cuvettes to the analyzer (Beckahm Coulter Access2) by the electrochemiluminescence method.

### Bone preparation

Tibias, after natural unfreezes, were deboned, weighed in a 0.01 g semi-analytical scale (Bel, model L 3102iH) and their lengths were measured by 150 mm stainless digital caliper (Lee tools 684132) and then, bone resistance analysis was proceeded, through the equipment (Ta.xt Plus -Texture Analyzer).

### Mineral analysis preparation

#### Decontamination of laboratory material

Before starting the laboratory procedures, a cleaning and adequate decontamination of materials used to determine the microminerals was carried out. All containers and glassware were washed with neutral detergent, then immersed in 10% nitric acid solution for 24 hours for decontamination, followed by washing in reverse osmosis water. Before use, the material was dried in an oven at 105°C [24].

#### Preparation of tibia samples

Tibias previously submitted to bone strength were used. After unfreezing, the bones were oven-dried at 105°C for 24 hours and then calcined in muffle for 4 hours at 600°C [25].

A 0.5 g sample was weighed on an analytical balance (± 0.0001 g) and digested with 6 ml of HNO3 (65% PA) for 10 minutes in the open system. After this interval, this mixture was diluted with 45 mL of purified water, to produce a final volume of 50 mL to determine the microminerals: Zn, Mn and Cu. The same sample after the first reading was again diluted with another 25 mL of water for the reading of the macro minerals Ca and P.

### Preparation of liver samples

After collection, livers were stored in plastic pots, identified and frozen at -20°C. Subsequently, the samples were unfrozen to room temperature and the livers were fractionated to increase the surface area of the fragment and obtain a homogeneous drying. The fractionated samples were oven-dried at 105°C for 24 h.

A fragment of approximately 0.5 g was weighed in an analytical balance (± 0.0001 g) and digested with 6.0 mL of HNO3 (65%) in a microwave oven (Mars Xpress: Technology Inside, CEM Corporation). The heating program used for the liver samples were: power of 1300, 1600 and 1600.

W in 1st, 2nd and 3rd stage; respectively. 10, 15 and 35 minutes in the 1st, 2nd and 3rd stages; respectively. The temperature was 120, 160 and 160°C in the 1st, 2nd and 3rd stages; respectively. After digestion, purified water was added to give a final volume of 25 ml.

#### Preparation of excreta and feed samples

The excreta were collected twice a day for 24 hours at the end of the experimental period. Sheets of Kraft paper were lined under the cages with spacing between them to avoid contamination. After collection, the samples were stored in identified sterile plastic bags and frozen at -20°C.

Subsequently, the excreta of the two samples were unfrozen, homogenized and pre-dried in a forced ventilation oven at 55°C for 72 hours and then crushed in a sterilized stainless-steel ball mill for two minutes. A 1 g sample was weighed on an analytical balance (± 0.0001g) and oven-dried at 105°C. Were weighed 0.5 g of samples in analytical balance (± 0.0001g) by adding 6 mL of 65% HNO3 and then submitted to microwave oven (Mars Xpress: Technology Inside, CEM Corporation), for complete digestion of the material. After digestion, the samples were diluted with reverse osmosis water to yield a final volume of 25 ml.

For feed analysis, the samples were collected after mixing and stored in identified plastic bags and frozen at -20°C for later analysis. The procedure applied to the feed samples was the same as that of the excreta, except for pre-drying, which was done in a forced ventilation oven.

### Quantification of trace minerals

The quantification of trace minerals (Zn, Mn and Cu) in the samples was obtained by optical emission spectrophotometry with inductively coupled plasma source (ICP-OES).

### Statistical analysis

The data were analyzed to test the assumptions of normality of errors and homoscedasticity of variance. Afterward, the data from performance, organ weight, mineral composition of the tibias, blood variables and liver were submitted to ANOVA and the means were compared by Student’s t-test, at 5% significance. The variables oviduct, bone breaking strength, complete blood count, progesterone, T4 and T3 were normalized using the Box-Cox transformation and monocytes by natural logarithm.

Statistical analysis was performed by the statistical software SAS^®^ version 9.2 [26]. To describe the EO and EM, the data were adjusted by Gompertz model with the following description:

$$\text{Y}=\alpha \;e^{-e-\beta (\text{x}-\tau )}$$

Where, Y is the dependent variable; α is the maximum EO or EM rate or the model asymptote; β is constant, x is the age in days; and τ is the inflection point (days); and *e* is Euler number 2.718281828.

#### Model identity analysis

Adjusted models for EO and EM were submitted to analysis of model identity and equality of parameters by the likelihood ratio test, using chi-square χ^2^ statistics, according to the methodology described by [27].

Subsequently, each parameter of the adjusted model was compared. The initial hypotheses are: A) H0: The equations are identical for both techniques, i.e. a common equation can be used as an estimate of the equations involved and B) H0: A given subset of parameters are the same in the sources.

## Results

Concerning hen’s development, no differences were observed in BW and BWG between the two groups of treatments, but, ADFI was significantly greater (P < 0.01) for birds that fed AACM (Table 4).

**Table 4:** Body weight (BW), body weight gain (BWG) and average daily feed intake of 182-day-old hens (26 weeks) fed diets containing different trace mineral sources.

Variables	BW (g)	BWG (g)	ADFI (g)
IM^1^	1687.81	570.59	99.48^b^
AACM^2^	1704.14	579.55	107.46^a^
MEANS	1695.98	575.07	103.47
*P* value	0.198	0.400	<0.001
SEM	6.29	5.26	1.17

^a, b^ Means followed by different letters in columns differ from each other by Student’s t-test (P≤0.05).

^1^Inorganic Mineral.

^2^AACM: Amino acids-mineral complexed; P = probability, SEM = Standard error of the mean.

**Fig 2:**
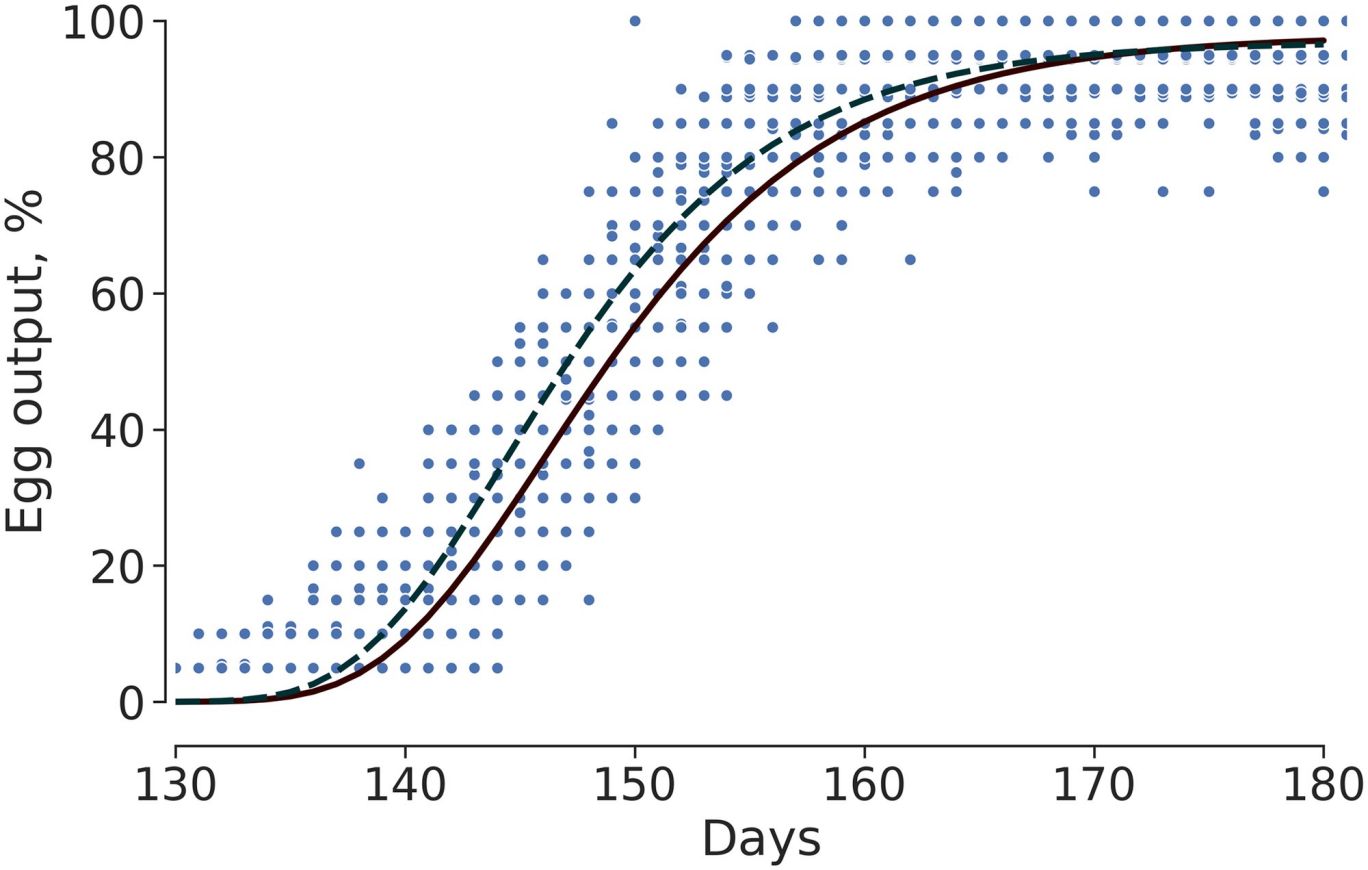
Daily egg output of laying hens supplemented different trace mineral sources:—Amino acids-mineral complexed (AACM); _____ Inorganic Mineral (IM); 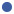 observed values.

Birds fed AACM reached 35% of production two days before (P = 0.04) the animals fed IM diet (Fig 2), shown by the parallelism test of Gompertz model for evaluation of EO. In Table 5 the significant difference for the parameter τ between the two treatments studied can be seen (*P* = 0.01).

**Table 5:** Analysis of egg output and egg mass of laying hens fed diets with different trace mineral sources.

Egg output			
Parameters of Gompertz model	α	β	τ
IM^1^	97.9281	0.1416	146.1
AACM^2^	96.9684	0.153	144.4
Identity model analysis for egg output			
	X2	GL	*P*-value
H0 = pm1 = pm2 = pm	1.56E+00	1	0.21
H0 = b1 = b2 = b	4.36E+00	1	0.04
H0 = t1 = t2 = t	9.33E+01	1	<0.001
w4:pm1 = pm2 e t1 = t2	9.58E+01	2	<0.001
w5: pm1 = pm2, b1 = b2 e c1 = c2	1.41E+02	3	<0.001
Egg mass			
Parameters of Gompertz model	α	β	τ
IM^1^	60.1756	0.132	147.7
AACM^2^	58.3943	0.1492	147.7
Identity model analysis of egg mass			
	X2	GL	*P*-value
H0 = pm1 = pm2 = pm	1.88E+05	1	0.171
H0 = b1 = b2 = b	1.78E+00	1	0.182
H0 = t1 = t2 = t	1.98E-02	2	0.888
w4:pm1 = pm2 e t1 = t2	2.33E+00	2	0.098
w5: pm1 = pm2, b1 = b2 e c1 = c2*	2.40E+00	3	0.066

^1^ IM: Inorganic mineral;

^2^ AACM: Amino acids-mineral complexed.

Y = α * and (-e (-β * (X- τ))); α = maximum egg output and egg mass; β = rate of egg mass; τ = inflection point in days;, e = Euler Number; X2:chi-square statistics; GL: Degree of freedom; X2:chi-square statistics; P- Probability.

**Fig 3:**
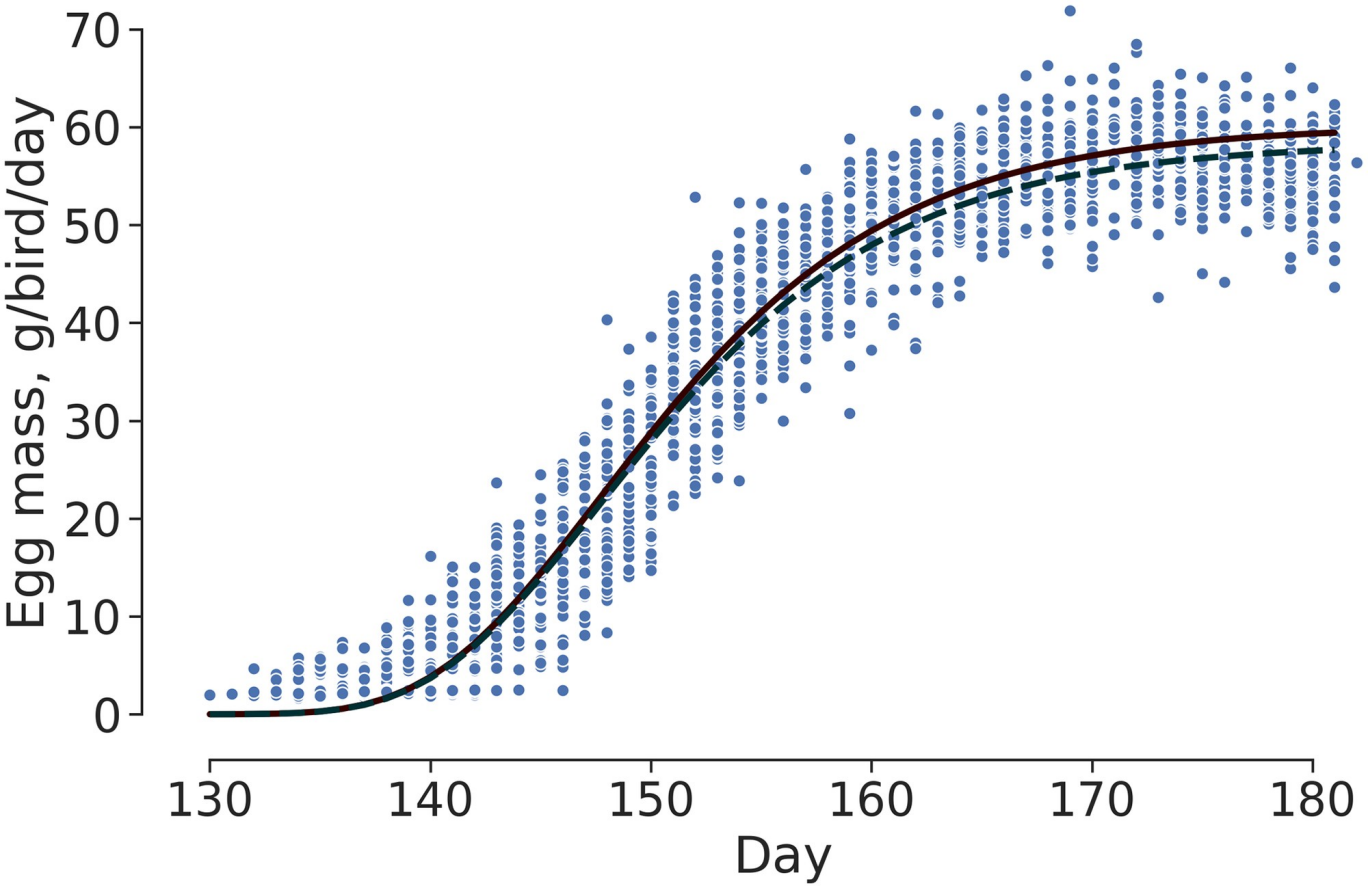
Daily egg mass of laying hens supplemented different trace mineral sources:—Amino acids-mineral complexed (AACM); _____ Inorganic Mineral (IM); 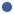 observed values.

The Gompertz model was also used for comparing daily EM between the two experimental groups (Table 5). No difference (P > 0.05) was observed between the models for the inflection point, showing that both treatments had the same EM throughout the experimental period (Fig 3).

No significant difference was found for liver (P = 0.95) and ovary weights (P = 0.76) between treatments. However, a significant difference was observed in the oviduct weight (P = 0.01) for the birds fed the diet composed of AACM, which presented heavier oviduct (Table 6).

**Table 6:** Liver, oviduct and ovary weight of 182-day-old hens (26 weeks) fed diets containing different trace mineral sources.

	Weight (g)		
Treatments	Liver	Oviduct	Ovary
IM	35.00	64.87^b^	41.13
AACM	35.07	73.54^a^	40.69
Average	35.04	69.20	40.91
*P* value	0.953	0.011	0.767
SEM	0.58	1.75	0.72

^a, b^ Means lacking a common superscript letter differ, *P* < 0.05 by Student’s t-test.

IM—Inorganic Mineral, AACM—Amino acids-mineral complexed, P = Probability, SEM = Mean Standard Error.

Hens fed AACM presented heavier tibia (*P* = 0.04) and no differences were observed in Seedor index (*P* = 0.34), bone resistance *(P* = 0.96), ash *(P* = 0.60) and length *(P* = 0.25) between the two groups of treatments (Table 7).

**Table 7:** Physical variables of the tibia of 182-day-old hens (26 weeks) in the peak of the egg production phase.

Treatments	Tibia weight (g)	Length (mm)	Seedor Index (mg mm^-1^)	Bone Breaking Strength (kgf/cm^2^)	Ash (%)	Ca (mg g^-1^)	P (mg g^-1^)
IM	10.17^b^	112.14	91.62	25.02	42.68	168.59	82.67
AACM	10.50^a^	112.67	92.85	25.10	43.21	169.65	83.30
Average	10.33	112.41	92.25	25.06	42.94	169.12	82.98
*P* value	0.036	0.249	0.337	0.957	0.603	0.90	0.884
SEM	0.79	0.23	0.64	0.73	0.49	4.03	2.02

^a^, ^b^ Means lacking a common superscript letter differ, P < 0.05 by Student’s t-test.

IM—Inorganic Mineral, AACM—Amino acids-mineral complexed; P = Probability, SEM = Mean Standard Error.

No significant difference was observed (P > 0.05) for the concentrations of Zn, Mn, Cu, Ca, P in the tibia and livers of hens (Table 8). Hens fed AACM excreted more Ca (*P* = 0.03), and also excreted a greater (*P* < 0.01) amount of Cu when compared to the birds that consumed Cu from the IM. Concerning the other minerals studied, no differences between the groups were observed.

**Table 8:** Mineral composition of the tibia, liver and excreta of 182-day-old hens (26 weeks) fed different trace mineral sources.

Treatments	**Liver**				
	Zn	Mn	Cu	Ca	P
	mg/kg			g/kg	
IM	122.38	12.94	17.16	0.738	15.06
AACM	129.60	12.86	17.62	0.648	15.16
Average	125.99	12.90	17.40	0.693	15.11
*P* value	0.572	0.947	0.687	0.069	0.895
SEM	6.166	0.580	0.553	0.024	0.378
	**Excreta**				
IM	311.59	311.96	39.72^b^	59.94^b^	8.24
AACM	318.19	319.59	46.55^a^	67.83^a^	8.75
Average	314.72	315.77	42.96	63.89	8.47
*P* value	0.354	0.412	0.004	0.029	0.228
SEM	3.44	4.51	1.27	1.85	0.21
	**Tibia**^1^				
IM	258.25	10.89	5.48	703.14	344.85
AACM	288.25	12.88	5.35	712.28	349.60
Average	273.25	11.88	5.41	707.71	347.22
*P* value	0.247	0.335	0.585	0.690	0.698
SEM	12.68	1.005	0.115	11.038	5.892

^a, b^ Means lacking a common superscript letter differ, *P* < 0.05 by Student’s t-test.

IM—Inorganic Mineral, AACM—Amino acids-mineral complexed; P = Probability, SEM = Mean Standard Error.

^1^g/kg tibia ash.

Table 9 shows blood variable results, in which hens fed AACM showed a greater level of red blood cells *(P* = 0.01) and white blood cell *(P* = 0.02), as well as tended to have greater hematocrit than the group of hens fed IM (*P* = 0.07). In the leukogram, the group fed AACM responded with greater (*P* = 0.006) serum levels of eosinophils and with greater response (P = 0.02) of heterophils. The AACM also tended to have greater levels of monocytes (*P* = 0.10), but they presented lower levels of lymphocytes (*P* < 0.01) than the group fed IM.

**Table 9:** Red blood cell (RBC), hemoglobin (HGB), hematocrit (HCT); mean corpuscular volume (MCV), mean corpuscular hemoglobin (MCH), complete blood count (CBC), white blood cell (WBC) and leukogram of 182-day-old hens (26 weeks) fed diets different trace mineral sources.

Treatments	**Blood Count**										
	RBC (10^6^ /mm^3^)	HGB (g %)		HTC (%)		MCV (fL)	MCH (%)		CBC (/mm^3^)		WBC (10^3^/mm^3^)
IM	2.35^b^	9.11		30.10		128.24	30.28		31.7		14.66^b^
AACM	2.45^a^	9.12		31.10		127.83	29.53		40.31		17.67^a^
Average	2.42	9.12		30.75		127.97	29.78		37.34		16.63
*P* value	0.012	0.930		0.071		0.814	0.224		0.241		0.02
SEM	0.021	0.083		0.26		0.81	0.29		3.32		0.62
Treatments	**Leukogram**										
	Heterophils		Eosinophils		Lymphocytes			Monocytes			
	%		%		%			%			
IM	34.22^b^		0.60^b^		60.11^a^			4.60			
AACM	42.31^a^		2.11^a^		48.0^b^			6.94			
Average	39.71		1.57		51.89			6.13			
*P* value	0.023		0.006		0.001			0.101			
SEM	1.69		0.27		1.85			0.66			

^a, b^ Means lacking a common superscript letter differ, P < 0.05 by Student’s t-test.

IM—Inorganic Mineral, AACM—Amino acids-mineral complexed; RBC—Red blood cell; HGB—Hemoglobin; HCT—Hematocrit; MCV—Mean Corpuscular Volume; MCH—Mean corpuscular hemoglobin; CBC—Complete blood count; WBC—White blood cell.; P = Probability, SEM = Mean Standard Error.

**Fig 4:**
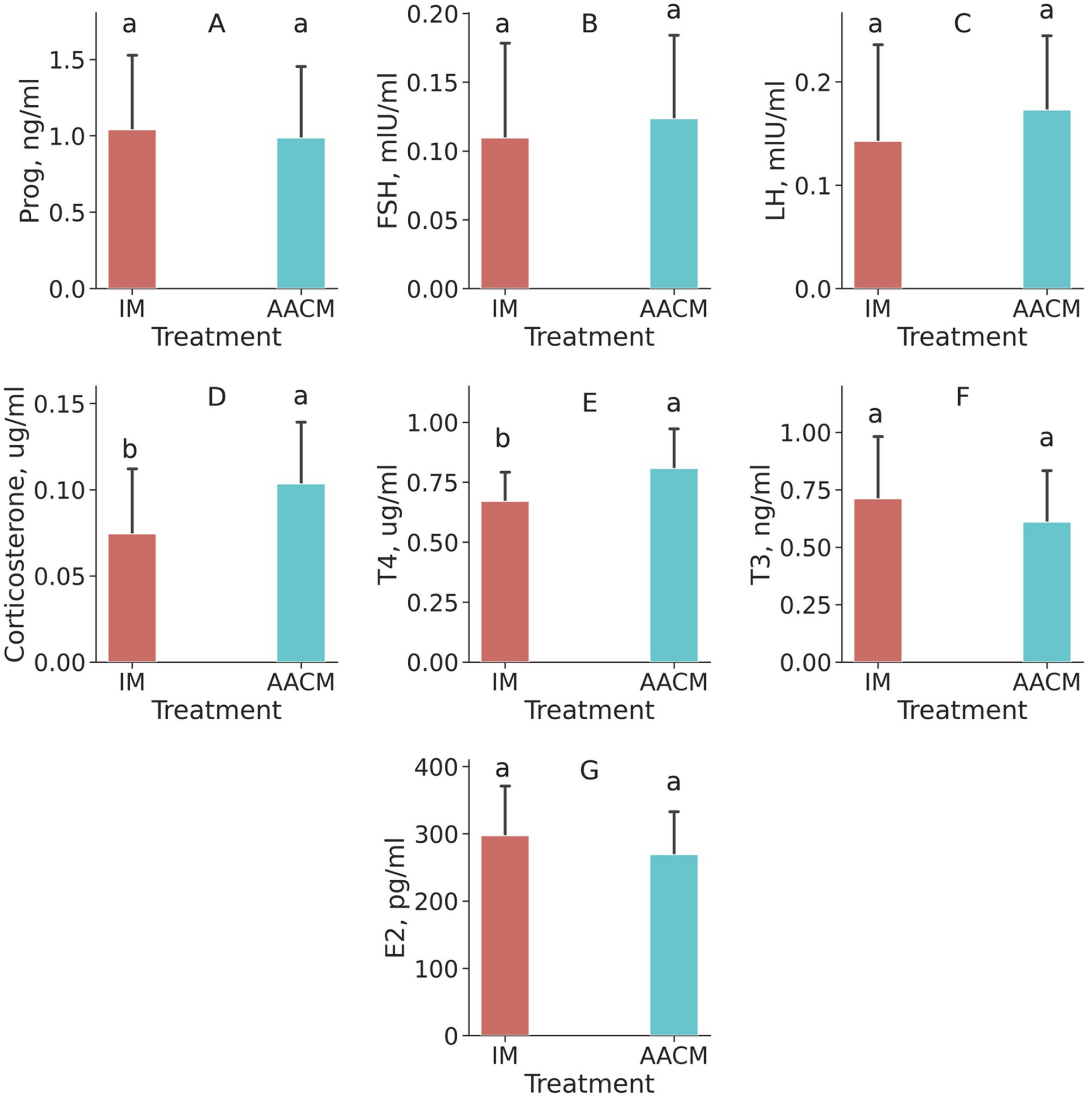
Hormonal variables of laying hens fed diets containing different trace mineral sources. A: Prog—progesterone; B: FSH—Follicle Stimulating Hormone; C: LH—Luteinizing Hormone; D: Cortisol; E: T4 –Total Thyroxine; F: T3 –Total Triiodothyronine; G: E2 –Estradiol; IM: Inorganic Mineral; AACM: Amino acids-mineral complexed. ^a, b^ Means lacking a common superscript letter differ, *P < 0*.*05*.

A greater serum concentration of T4 was observed in the group of hens fed AACM (*P* = 0.05). This group also tended to present a greater level of corticosterone (*P* = 0.056). Nevertheless, no difference was observed for T3, FSH, progesterone, estradiol and LH (Fig 4).

## Discussion

In this study, the supplementation with AACM promoted better oviduct development, precocity, tibial weight, immune response and higher secretion of T4; an indispensable hormone to reproduction functions.

The greater weight of the oviduct found in birds that consumed AACM justifies the precocity of laying, compared to the IM group. In fact, birds with greater weight of the oviduct are desirable. After all, albumen and shell are formed in the oviduct, and a great proportion of EW is conferred by the albumen content [28]. Growth Hormone (GH) is linked to the growth of this organ [29], as this hormone is responsible for the expression of the genes of some specific proteins and the reduction of apoptotic cells. Moreover, the increased availability of Mn from the AACM treatment birds may have conferred with increased production and GH release as well as circulating insulin, since Mn is actively involved in the production of these hormones. In fact [30], demonstrated that Mn deficient diets decrease the release of insulin, GH and IGF-1. Zn deficiency also results in reduced IGF-I concentration [31, 32]. However, Cu is considered the major trace mineral determinant in the release of IGF-I [32]. The results found in this study, in part, are consistent with the found by Medeiros et al. [33] where the authors observed increased cell integrity of the oviduct for birds that fed chelated Zn, Se and Mn.

Concerning the EO curve, the hens fed AACM diet reached the inflection point of the Gompertz model two days before the hens fed IM. This variable was influenced by the greater development of the oviduct leading to a better physiological development by preparing the birds earlier for the laying phase. Hens fed AACM-containing diets also presented longer intestines, which probably contributed to increasing the area of nutrients absorption and thus, a better development of reproductive organs, leading to greater precocity. A study carried by [34] showed that birds fed diets containing Zn AACM have higher villus growth, villus:crypt ratio and surface area of absorption. Shao et al. [35] reported that Zn also promotes reduction of apoptosis and increased cell proliferation and consequently increases the absorptive capacity of the gastrointestinal tract.

The difference in tibial weight of the birds fed AACM-containing diet may be related to the high bone mineralization [36, 37]. It might mean that hens with better bone mineralization during the start of the laying phase will produce better eggshells for a longer period of productive life.

The greater Cu excretion for birds fed AACM-containing diet, observed in this study, is controversial. Gao et al. [19] showed that all the AACM forms of Cu are better absorbed than copper sulfate. Considering this, we can speculate, it is possible that due the association of the two sources, more Cu resulting from uptake by inorganic receptors has been retained as metallothionein storage in the enterocytes, and as a result of normal enterocytes apoptosis, it was excreted.

The values of the blood variables for both treatments were within normal range [38]. However, the greater bioavailability AACM in the organism provided a better immunological status and the birds were more prepared to the challenges they were exposed to, presenting a greater serum concentration of red blood cells, eosinophils and total leucocytes, which also tended to present greater hematocrit and monocytes, and reduced levels of lymphocytes. Hens were reared under heat stress natural conditions and high- density cages, as it is common in any industrial environment. Looking for the blood cells count, it is possible to state that hens fed AACM had a remarkable immune response, which was an expected response since it is known that Zn, Mn and Cu are closely involved with the immune system [39, 40].

The supplementation of layer-type pullets with Zn, Mn and Cu amino acid complexes, since the first day of age, promoted longer intestines and greater hormonal secretion with consequently greater development of oviduct and heavier tibia at the peak production phase.
